# Local Synthesis of CaMKII Does Not Contribute to Activity-Dependent Generation of Ghost Boutons

**DOI:** 10.1523/ENEURO.0196-26.2026

**Published:** 2026-09-11

**Authors:** Kelsey J. Clements, Leslie C. Griffith

**Affiliations:** Department of Biology and Volen Center for Complex Systems, Brandeis University, Waltham, Massachusetts 02254-9110

**Keywords:** calcium/calmodulin-dependent protein kinase II, *Drosophila*, local translation, synapse formation

## Abstract

Activity-dependent structural plasticity is essential for the growth and remodeling of synaptic connections. At the *Drosophila melanogaster* larval neuromuscular junction (NMJ), spaced stimulation induces the translation-dependent formation of ghost boutons (GBs), which are immature boutons that lack a corresponding postsynaptic structure. Calcium/calmodulin-dependent protein kinase II (CaMKII) has previously been implicated in GB formation and is locally translated at synapses, raising the possibility that activity-dependent CaMKII synthesis contributes to GB formation. We examined activity-induced synaptic outgrowth in female larvae using a spaced depolarization paradigm combined with genetic manipulations of the endogenous CaMKII locus. While whole-animal *CaMKII* null mutants exhibited fewer GBs after spaced depolarization, selective disruption of activity-dependent CaMKII synthesis by deletion of the *CaMKII* 3′ untranslated region (3′UTR) in either presynaptic motor neurons or postsynaptic muscle cells had no effect on GB formation. Consistent with this, inhibition of the signaling pathways upstream of CaMKII synthesis also did not impair bouton outgrowth. Cell-specific deletion of the CaMKII coding region in presynaptic neurons also did not alter GB formation, but postsynaptic deletion significantly reduced it. These findings demonstrate that local synthesis of CaMKII is dispensable for activity-dependent GB formation. Instead, our results indicate that only steady-state CaMKII protein is necessary for this specific type of structural plasticity and identifies a new transsynaptic function for postsynaptic CaMKII in GB formation. These findings distinguish the role of CaMKII protein from CaMKII synthesis and suggest that other locally translated proteins underlie the protein synthesis dependence of GB formation.

## Significance Statement

Local protein synthesis is thought to support long-lasting forms of synaptic plasticity. At the *Drosophila* neuromuscular junction, CaMKII is synthesized during patterned stimulation, which also triggers CaMKII-dependent ghost bouton (GB) formation, suggesting CaMKII local synthesis might be involved in this form of structural plasticity. In contrast to this hypothesis, disrupting activity-dependent CaMKII translation in either presynaptic or postsynaptic cells had no effect on GB formation. Instead, loss of CaMKII protein in the whole animal blocked synaptic outgrowth, and loss of postsynaptic CaMKII protein reduced it. Our findings indicate that CaMKII protein function, but not local CaMKII synthesis, is involved in activity-dependent GB formation. Identifying the precise molecular players driving local translation-dependent remodeling remains a task for future research.

## Introduction

The *Drosophila* larval neuromuscular junction (NMJ) is a classic model for understanding the molecular basis of synapse formation (Menon et al., 2013). As an excitatory glutamatergic synapse, it has strong molecular, functional, and structural similarities to vertebrate excitatory central synapses, and studies at the fly NMJ have yielded insights that have informed our understanding of mammalian synapse formation. Because growth at this developmental stage is rapid, with body mass increasing ca. 200-fold over 4 d (Tennessen and Thummel, 2011), matching motor neuron input to muscle area requires fast, tightly regulated processes for synapse addition.

Addition of synapses at the NMJ is initiated by blebbing of the presynaptic axon (Fernandes et al., 2023) with subsequent cytoskeletal rearrangements (Ataman et al., 2008; Piccioli and Littleton, 2014). The initial immature contact of the presynaptic axon with the muscle can contain synaptic vesicles but is typically devoid of mature active zone complexes. At this stage, the immature synapse also lacks the postsynaptic components that define mature synapses: DLG, a synaptic scaffold homologous to mammalian PSD-95, and clustered glutamate receptors. Once formed, these structures can either be retracted and cleared (Fuentes-Medel et al., 2009) or can undergo Wnt- and/or BMP-dependent maturation, acquiring postsynaptic scaffolding and effectors and mature active zones (Ataman et al., 2006; Piccioli and Littleton, 2014).

While new bouton formation can be seen in intact animals (Zito et al., 1999) and unstimulated larval fillets (Piccioli and Littleton, 2014), the number of new boutons initiated is substantially increased by neuronal activity (Ataman et al., 2008; Piccioli and Littleton, 2014; Fernandes et al., 2023). In the first report of activity-dependent sprouting (Ataman et al., 2008), live imaging revealed thin protrusions (“synaptopods”) immediately on stimulation, but these structures were typically lost upon fixation. After ∼2 h stimulation, larger, rounder presynaptic protrusions could be visualized in fixed tissue, and the term “ghost bouton” (GB) was coined to describe these more stable structures. Both types of protrusions lacked postsynaptic scaffolding and receptors, but the appearance of GBs in fixed tissue at longer time points required multiple spaced stimuli and was blocked by protein synthesis inhibitors. In this study, we focus exclusively on this more stable protein synthesis-dependent population of GBs.

The molecular pathways underlying the formation of protein synthesis-dependent GBs include both pre- and postsynaptic players. Postsynaptic Wg/Wnt signaling via GRIP-dependent nuclear signaling by DFz-2/Frizzled-2 is required (Ataman et al., 2006, 2008). Retrograde BMP signaling to reorganize presynaptic actin networks is also important (Piccioli and Littleton, 2014). Presynaptically, kinases such as Shaggy/GSK-3β, LimK, PKA, and Akt have been implicated as regulators, as has the PKA-dependent redistribution of synaptic vesicles by synapsin (Piccioli and Littleton, 2014). However, while it is clear these pathways are required to generate morphological changes in response to activity, new protein synthesis has not been shown to regulate their mobilization. Engagement of these cascades relies rather on changes in localization and/or phosphorylation of preexisting components. The identity of the protein synthesis-dependent factor required for GB formation has not been determined.

One potential candidate is CaMKII, a protein known to be locally synthesized in both mammals (Mayford et al., 1996) and *Drosophila* (Hillebrand et al., 2010). CaMKII has multiple roles at the larval NMJ and contributes to presynaptic plasticity (Carrillo et al., 2010; Chun-Jen Lin et al., 2011; Shakiryanova et al., 2011), postsynaptic organization (Koh et al., 1999; Beumer et al., 2002), and retrograde muscle-to-neuron signaling (Haghighi et al., 2003; Kazama et al., 2003; Morimoto et al., 2010; Newman et al., 2017). Manipulating CaMKII can also alter the number of mature boutons, i.e., synapses with presynaptic active zones and scaffolded postsynaptic receptors (Wang et al., 1994; Koh et al., 1999; Kazama et al., 2003; Morimoto-Tanifuji et al., 2004; Carrillo et al., 2010). These studies have largely focused on the role of the kinase in maturation and stabilization of new contacts, including recruitment of the DLG scaffold and organization of the postsynaptic receptor population, rather than on the initial formation of GBs.

At the larval NMJ, spaced depolarization can increase synaptic CaMKII levels in concert with GB formation, and this increase is blocked by cycloheximide (Nesler et al., 2016). A linkage of these two phenomena was suggested by experiments in which presynaptic CaMKII RNAi was shown to decrease GB formation. However, because RNAi lowers basal CaMKII levels as well as the magnitude of activity-induced synthesis, these experiments could not distinguish whether GB formation requires newly synthesized CaMKII or simply an adequate pool of preexisting kinase. Subsequent studies have also determined that patterned activity can increase CaMKII in both the pre- and postsynaptic compartments (Clements et al., 2025), expanding the potential locus of action of newly synthesized CaMKII in GB formation. Because *CaMKII* is an essential gene (Kuklin et al., 2017), all the previous GB studies were done using pharmacology or transgenic tools rather than manipulations of the endogenous *CaMKII* locus.

In recent years, CRISPR and cell-specific recombination systems have enabled precise manipulations of the endogenous locus of genes of interest, providing the ability to alter gene function in a more nuanced manner. Using a set of such tools for *CaMKII*, we reexamined the role of this kinase in GB formation. We find that *CaMKII* null animals, which have a severe global reduction in CaMKII, have attenuated GB formation. In contrast, selectively blocking local translation of CaMKII in either the pre- or postsynaptic compartment does not impair stimulation-induced GB formation. Cell-specific deletion of the *CaMKII* coding region indicates that initial formation of new boutons relies on the presence of postsynaptic CaMKII protein. Together, these findings demonstrate that CaMKII protein is required for activity-dependent bouton formation, but acute local synthesis of CaMKII is not.

## Materials and Methods

### Drosophila culture

*Drosophila* stocks were reared at 25°C in standard cornmeal dextrose medium and checked regularly for mites and contamination. Crosses for experiments consisted of three males and three virgin females and were transferred to new vials every 2 d to ensure the vials did not get overcrowded. Frt-Stop-Frt-EGFP::CaMKII, Frt-3′UTR-Frt and Frt-Coding-Frt flies, *CaMKII* null (frt-stop-frt-Halo-CaMKII), T287T, T287A and T287A, T306/7AA flies were generated as previously described (Chen et al., 2022). Frt-3′UTR-Frt flies have Frt sites flanking the entire long isoform of the *CaMKII* 3′UTR, and Frt-Coding-Frt flies have Frt sites flanking the region of the *CaMKII* locus corresponding to the catalytic region. Genotypes for each type of larvae used in experiments are as follows:

Flp DelUTR Ctrl: UAS-Flp/+; Frt-3′UTR-Frt/Frt-3′UTR-Frt
C164 DelUTR Ctrl: C164-Gal4/+; Frt-3′UTR-Frt/Frt-3′UTR-Frt
MHC DelUTR Ctrl: MHC-Gal4/+; Frt-3′UTR-Frt/Frt-3′UTR-Frt
Pre DelUTR: C164-Gal4/UAS-Flp; Frt-3′UTR-Frt/Frt-3′UTR-Frt
Post DelUTR: MHC-Gal4/UAS-Flp; Frt-3′UTR-Frt/Frt-3′UTR-Frt
Flp DelCoding Ctrl: UAS-Flp/+; Frt-Coding-Frt/Frt-Coding-Frt
C164 DelCoding Ctrl: C164-Gal4/+; Frt-Coding-Frt/Frt-Coding-Frt
MHC DelCoding Ctrl: MHC-Gal4/+; Frt-Coding-Frt/Frt-Coding-Frt
Pre DelCoding: C164-Gal4/UAS-Flp; Frt-Coding-Frt/Frt-Coding-Frt
Post DelCoding: MHC-Gal4/UAS-Flp; Frt-Coding-Frt/Frt-Coding-Frt
CaMKII null mutant −/− : Frt-stop-Frt-Halo-CaMKII/ Frt-stop-Frt-Halo-CaMKII
CaMKII heterozygous mutant +/−: Frt-stop-Frt-Halo-CaMKII/+

### Spaced depolarization/5× high-potassium experiments

Wandering third instar female larvae were used in all experiments to ensure they were in the same developmental stage. Larvae were dissected in cold HL3.1 and left semistretched so that the muscles could contract without damage. The CNS was checked to confirm there was no damage to the ventral nerve cord (VNC) or axons, unless an axotomy was conducted by severing the axon near the VNC with Vannas scissors. Spaced depolarization/high-potassium stimulation was conducted as previously described (Ataman et al., 2008), and the osmolarity of both the normal and high K+ solution was verified to be within 5 mOsm of each other. For Figure 5*A*, larvae were incubated for 15 min in 100 nM Wortmannin (MilliporeSigma) before stimulation, and Wortmannin was added to both normal and high K+ solutions. At the end of the stimulation protocol, the larval fillets were fixed with 4% PFA in HL3.1 for 25 min. PFA was removed by washing three times for 10 min in PBX (PBS with 0.1% Triton X-100). Fillets were incubated with the primary antibody in PBX with 10% Normal Goat Serum overnight at 4°C, washed three times for 10 min in PBX, and then incubated for 1 h at room temperature with the secondary antibody in PBX. The fillets were then washed three times for 10 min in PBX and mounted in SlowFade Diamond mounting media (Thermo Fisher Scientific). Antibody dilutions and manufacturers are as follows: Rabbit Anti-Dlg (1:500, Boster Bio), Mouse Anti-CaMKII (1:1,000, Cosmo Bio), Goat Anti-Rabbit Alexa Fluor 488 (1:250, Thermo Fisher Scientific), Goat Anti-Rabbit Alexa Fluor 555 (1:250, Thermo Fisher Scientific), and Goat anti-Horseradish Peroxidase Alexa Fluor 647 (1:250, Jackson ImmunoResearch).

### Microscopy and image analysis

NMJs were imaged on a Zeiss LSM880 confocal microscope in the Brandeis Light Microscopy Core Facility. The NMJs from segments A3 and A4 of muscle 6/7 were imaged from each larva using the 488, 561, and 633 lasers and the 63× objective. *Z* stacks were acquired with a 0.5 µM step throughout the entire *Z* axis of the NMJ. After imaging, a maximum intensity projection was created in FIJI/ImageJ, and the Hrp and Dlg channels merged. Boutons were counted as GBs if they were positive for Hrp but lacked the corresponding Dlg ring. For experiments in which the intensity of CaMKII signal was analyzed, the Hrp or Dlg channel was used to create a mask of the presynaptic or postsynaptic region, which was used to quantify the fluorescent intensity of the presynaptic or postsynaptic region. The experimenter performed the analyses blind to the conditions. Data were analyzed in GraphPad Prism using the appropriate statistical tests (for statistical analyses, see Extended Data Fig. 1-1).

**Figure 1.**
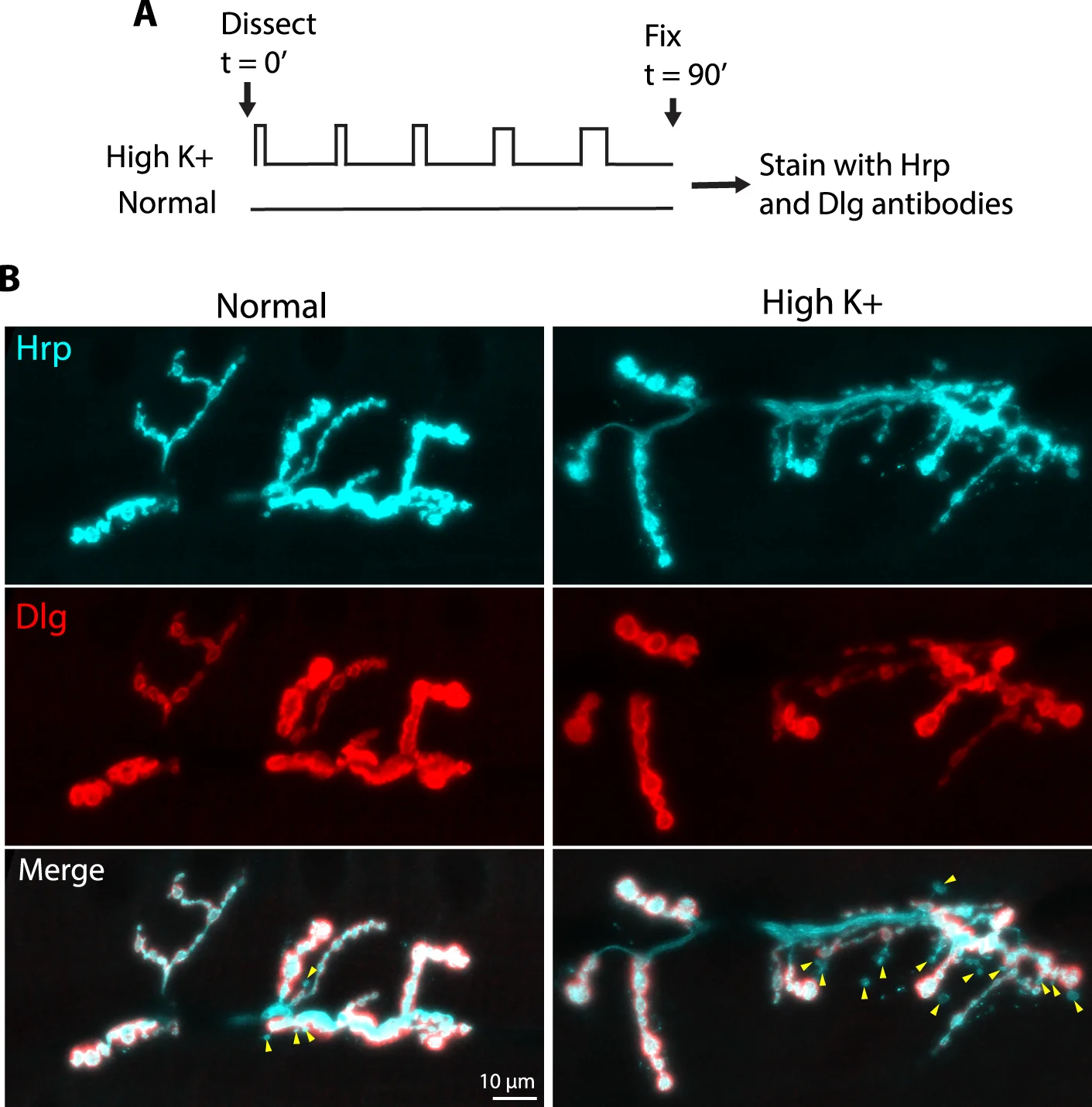
Experimental design and analysis. ***A***, Schematic diagram demonstrating the high K+ spaced depolarization protocol. Larvae were dissected and exposed to pulses of a high K^+^ solution with rest periods in normal HL3.1 saline. Controls were only exposed to normal HL3.1. Larvae were fixed after the protocol and stained with Hrp (presynaptic) and Dlg (postsynaptic). ***B***, Representative images of NMJs showing enhancement of GB formation in high K^+^ stimulated larvae. GBs (yellow arrowheads) were identified by counting Hrp+ (cyan) and Dlg− (red) synaptic protrusions.

10.1523/ENEURO.0196-26.2026.f1-1 Figure 1-1 Statistical analyses and genotypes. Download Figure 1-1, DOCX file.

## Results

CaMKII is locally synthesized at synapses both in mammals (Mayford et al., 1996) and in *Drosophila* (Hillebrand et al., 2010; Nesler et al., 2016; Chen et al., 2022; Clements et al., 2025). Formation of GBs at the larval NMJ is dependent on protein synthesis (Ataman et al., 2008) and has also been shown to be blocked by both presynaptic RNAi knockdown and inhibition of CaMKII (Nesler et al., 2016). We were interested in determining if these two sets of observations reflected a specific requirement for the local synaptic synthesis of presynaptic CaMKII or whether reduction of steady-state CaMKII activity alone was sufficient to block GB formation. To do this, we utilized a spaced depolarization protocol described in Ataman et al. (2008), in which NMJs from third instar larvae are exposed to pulses of a high-potassium depolarizing solution, with rest periods in normal HL3.1 saline. Unstimulated larvae were exposed to normal HL3.1 saline the entire time (Fig. 1).

**Figure 2.**
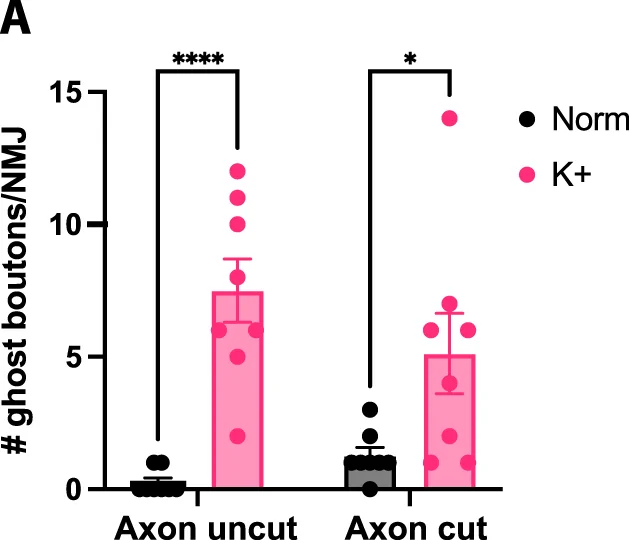
Local factors are sufficient for activity-dependent GB formation. ***A***, Larvae were dissected and the axons were cut to one side of the body wall before spaced depolarization. Axotomized NMJs produced GBs when stimulated. Groups were analyzed with a two-way ANOVA with Šídák's multiple-comparisons test. ****p = <0.0001, *p = 0.0369. *n* = 8 NMJs.

We first addressed the issue of whether GB formation is a local event or whether it requires input from the central nervous system, via either nerve activity or factors that are trafficked from the motor neuron cell body. Before applying the spaced depolarization protocol, we cut the motor nerve at the NMJ from the ventral nerve cord on one side of the filet and assessed GB formation on the axotomized versus intact sides. Figure 2 shows that communication from the ventral nerve cord is not required for GB formation; local factors are sufficient to drive the sprouting response to activity.

**Figure 3.**
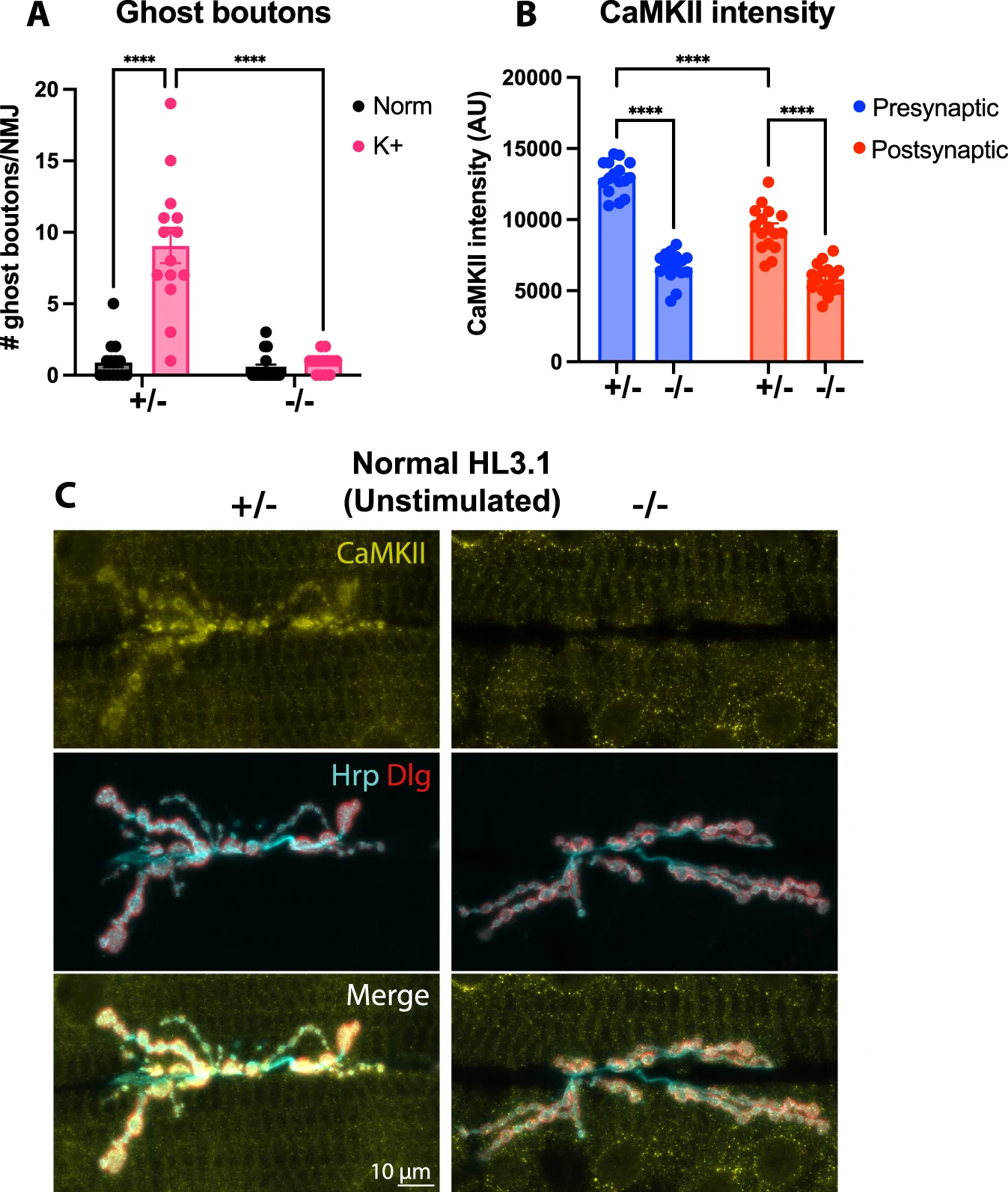
Synaptic CaMKII is required for GB formation. ***A***, Number of GBs formed in heterozygous and homozygous CaMKII null mutants. GB formation is blocked in homozygous nulls. ***B***, Quantification of CaMKII intensity in unstimulated NMJs from ***A***. ***C***, Representative images from NMJs analyzed in ***B***. Groups were analyzed with a two-way ANOVA with Šídák's multiple-comparisons test. ****p = <0.0001. *n* = 13–16 NMJs.

We next asked if GB formation was impaired in a *CaMKII* null mutant. These mutants develop morphologically normal-looking synapses and survive into the third instar due to residual maternal *CaMKII* mRNA (Kuklin et al., 2017). Despite having normal-looking NMJs, synaptic kinase levels are reduced in the mutant because the maternal mRNA lacks distal 3′UTR sequences that support local translation, which is required for synaptic enrichment of axonal CaMKII (Chen et al., 2022). Mutants lacking the *CaMKII* 3′UTR are also unable to increase CaMKII levels in response to activity (Clements et al., 2025). Figure 3*A* shows that GB formation in *CaMKII* null mutants is decreased, consistent with published results using RNAi and inhibitors. However, because CaMKII levels are reduced on both sides of the synapse (Fig. 3*B*,*C*), this result cannot distinguish whether the CaMKII requirement is pre- or postsynaptic or provide a clean distinction between a requirement for some steady-state level of CaMKII activity versus a requirement for new synthesis of CaMKII.

**Figure 4.**
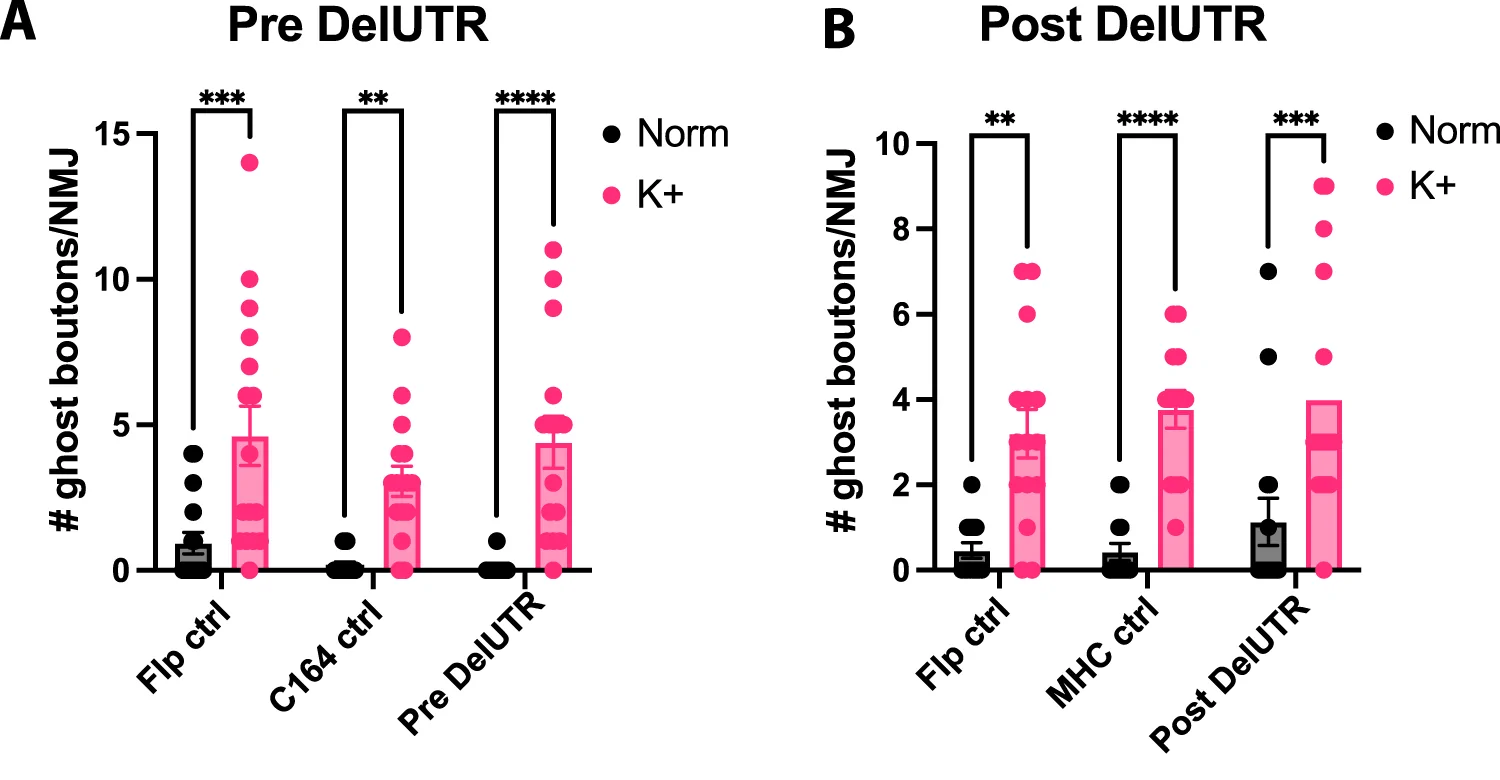
Neither presynaptic nor postsynaptic CaMKII synthesis is needed for GB formation. The 3′UTR of *CaMKII* was deleted using presynaptic-specific (C164-Gal4, ***A***) or postsynaptic-specific (MHC-Gal4, ***B***) expression of Flp recombinase. Stimulated NMJs showed induction of GB formation in all genotypes, indicating 3′UTR-mediated CaMKII synthesis is not required for GB formation. Groups were analyzed with a two-way ANOVA with Šídák's multiple-comparisons test. ***A***, ***p = 0.0002, **p = 0.0057, ****p = <0.0001. *n* = 15–16 NMJs. ***B***, **p = 0.0014, ****p = <0.0001, ***p = 0.0003. *n* = 13–16 NMJs. For data on CaMKII protein levels, see Extended Data Figure 4-1.

Recent work has defined a molecular pathway underlying presynaptic local CaMKII synthesis (Clements et al., 2025). To more directly address the role of presynaptic activity-dependent synthesis of CaMKII, we asked if perturbation of elements supporting local CaMKII synthesis would disrupt GB formation. We first looked at the 3′UTR of the *CaMKII* mRNA using animals in which the entire 3′UTR of the *CaMKII* gene could be deleted by cell-specific expression of Flp recombinase. Remarkably, removal of the 3′UTR only in presynaptic cells, which blocks activity-dependent CaMKII synthesis in axons (Chen et al., 2022; Clements et al., 2025), did not alter the ability of spaced depolarization to increase the number of GBs (Fig. 4*A*).

10.1523/ENEURO.0196-26.2026.f4.1 Figure 4-1 **NMJs lacking the 3'UTR of CaMKII in the postsynaptic muscle have reduced activity-dependent CaMKII synthesis.** After spaced depolarization, larvae were fixed and stained with anti-CaMKII, anti-Dlg, and anti-Hrp antibody. A) Representative images of postsynaptic CaMKII protein in NMJs lacking the 3'UTR in the postsynaptic region. The area within the dotted lines indicate the postsynaptic region. B) Quantification of CaMKII protein intensity represented in panel A. Analyzed with a 2 Way ANOVA with Šídák's multiple comparisons test B: ** p= 0.0011 Flp ctrl Norm vs. K+, **p = 0.0026 MHC ctrl Norm vs K+. n = 10-12 NMJs. Download Figure 4-1, TIF file.

Since the NMJ displays robust transsynaptic signaling, both from the motor neuron to muscle, but also from the muscle to neuron, we also examined whether postsynaptic protein synthesis may be involved in GB formation. We generated animals lacking the 3′UTR in the postsynaptic cells. This manipulation reduces steady-state postsynaptic CaMKII levels and blocks activity-stimulated postsynaptic CaMKII synthesis (Extended Data Fig. 4-1). NMJs from these larvae displayed normal high-potassium-induced GB formation (Fig. 4*B*), confirming that neither pre- nor postsynaptic activity-dependent CaMKII synthesis is necessary for activity-stimulated synaptic outgrowth.

**Figure 5.**
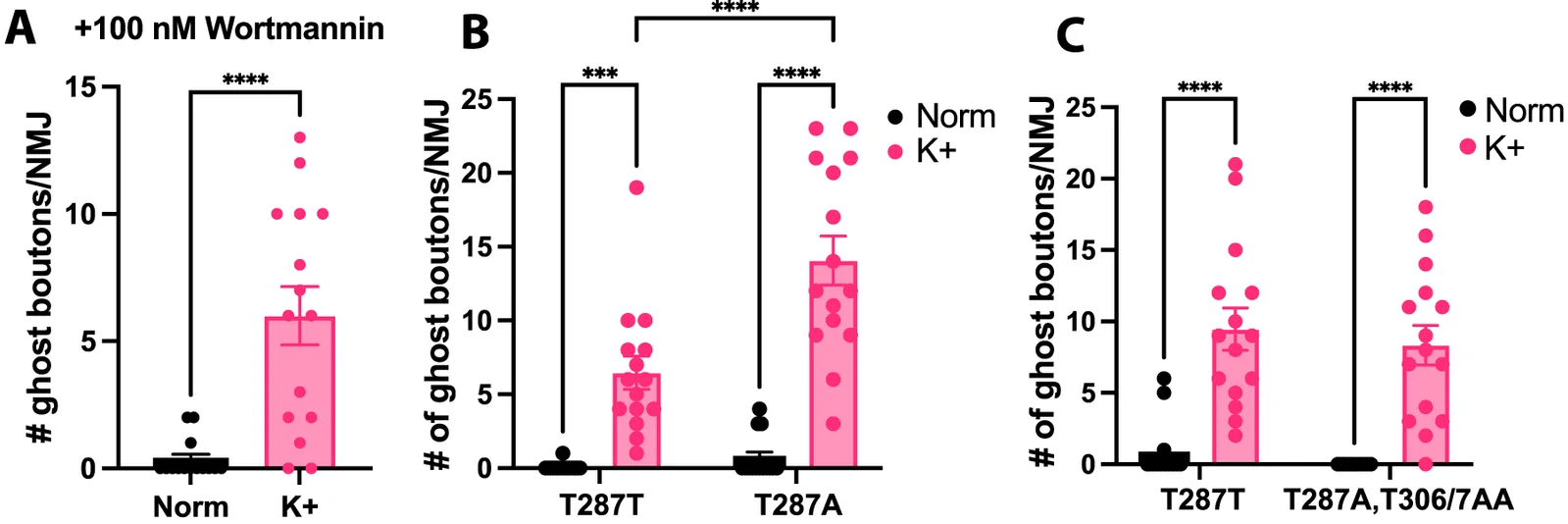
GB formation is independent of the molecular pathway underlying activity-dependent local CaMKII synthesis. ***A***, Pi3K does not affect GB formation. Larvae were treated with 100 nM Wortmannin in both normal and high K^+^ solutions. ***B***, ***C***, Control larvae (T287T) or T287 phosphorylation-deficient larvae (T287A or T287A, T306/7AA) all demonstrated normal GB formation, with T287A NMJs showing enhanced GB formation. ***A***, Analyzed with a two-tailed Mann–Whitney test. ****p = <0.0001. *n* = 14,15 NMJs. ***B***, ***C***, Analyzed with a two-way ANOVA with Šídák's multiple-comparisons test. ***B***, ***p = 0.0002, ****p = <0.0001. *n* = 14–16 NMJs. ***C***, ****p = <0.0001. *n* = 14–16 NMJs.

We next interrogated the signaling machinery underlying CaMKII local synthesis. Previous work has found that CaMKII autophosphorylation activates PI3K/Akt/mTor signaling to stimulate activity-dependent CaMKII synthesis. Inhibition of PI3K with Wortmannin, a manipulation that blocks activity-dependent CaMKII increases (Clements et al., 2025), failed to block stimulation of GB formation (Fig. 5*A*). To test the role of CaMKII autophosphorylation, which is upstream of the PI3K pathway at the NMJ (Chun-Jen Lin et al., 2011), we utilized larvae in which the endogenous T287 site is mutated to alanine, blocking autophosphorylation in all cells. The *CaMKII^T287A^* larvae have previously been shown to be deficient in both phosphorylation of Akt and CaMKII synthesis (Clements et al., 2025). In these animals, GB formation was normal (Fig. 5*B*,*C*), confirming that blocking the signaling upstream of activity-dependent CaMKII synthesis has no effect on GB formation.

**Figure 6.**
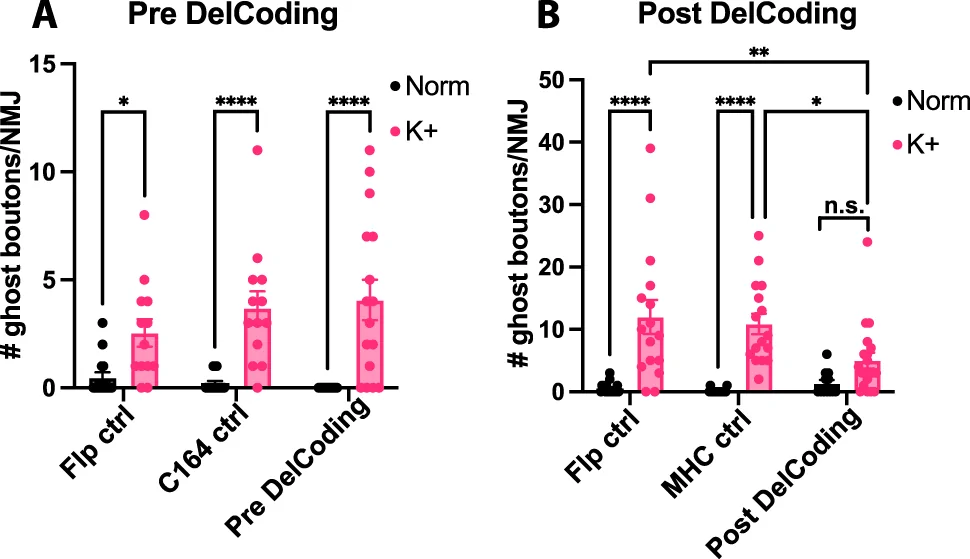
Depletion of postsynaptic, but not presynaptic CaMKII protein reduces GB formation. ***A***, ***B***, The coding region of *CaMKII* was deleted by presynaptic (***A***) or postsynaptic (***B***) specific expression of Flp recombinase. Presynaptic DelCoding NMJs had normal activity-dependent GB formation, while stimulated postsynaptic DelCoding showed blunted GB formation. Analyzed with a two-way ANOVA with Šídák's multiple-comparisons test. ***A***, ***p = 0.0003, ****p = <0.0001. *n* = 13–16 NMJs. ***B***, ****p = <0.0001, **p = 0.0023, *p = 0.0148. *n* = 16–20 NMJs. For data on CaMKII protein levels, see Extended Data Figure 6-1.

These results suggest that new translation of CaMKII is not required for GB formation, but they do not contradict the idea that some baseline level of presynaptic CaMKII may be necessary. While the presynaptic DelUTR animals have lower levels of synaptic CaMKII in motor neuron terminals, they likely have normal levels of CaMKII in the soma since the 3′UTR is not required to set somatic CaMKII levels (Chen et al., 2022). To determine whether the CaMKII protein in the neuron soma is compensating for the lack of synaptic protein, we examined GB formation in animals with the coding region of CaMKII removed using presynaptic-specific Flp-Frt recombination, a manipulation which reduces CaMKII levels substantially and blocks activity-dependent increases in kinase (Extended Data Fig. 6-1). In contrast to published results using depletion of presynaptic CaMKII protein with RNAi, which blocked GB formation (Nesler et al., 2016), the number of GB formed during spaced depolarization in NMJs from these presynaptic DelCoding animals was not significantly different from controls (Fig. 6*A*). To determine whether CaMKII-dependent retrograde signaling from the postsynaptic region was affecting GB formation, we also deleted the CaMKII coding region from the muscle cell, which also reduces basal CaMKII and blocks new synthesis (Extended Data Fig. 6-1). Interestingly, NMJs from these animals produced significantly fewer GBs compared with genetic controls (Fig. 6*B*), indicating a role for postsynaptic or muscle CaMKII protein in GB formation.

10.1523/ENEURO.0196-26.2026.f6-1 Figure 6-1 **NMJs lacking the coding region of CaMKII have reduced steady state and activity-dependent levels of CaMKII protein.** Larvae were prepared as described in extended figure 4.1. A,C) Representative images of presynaptic (A) or postsynaptic (C) CaMKII protein in NMJs lacking the CaMKII coding sequence in the presynaptic (A) or postsynaptic (C) region. Area within the dotted line indicates the presynaptic region in (A) and the area within the two dotted lines indicates the postsynaptic region in (C). B ,D) Quantification of presynaptic (B) or postsynaptic (D) CaMKII protein intensity represented in panels A and C. Analyzed with a 2 Way ANOVA with Šídák's multiple comparisons test. B: **** p =<0.0001, *** p = 0.0004, ** p = 0.0046. N = 11-16 NMJs. D: **** p = <0.0001, *** p = 0.0003. N = 10-15 NMJs. Download Figure 6-1, TIF file.

## Discussion

CaMKII local synthesis has been theorized to be involved in the stabilization of behavioral and synaptic plasticity. Several lines of evidence have supported this idea, including the dependence of the late phase of LTP and memory on the presence of the 3′UTR (Miller et al., 2002; Steward, 2002). Since removal of the 3′UTR typically also decreases steady-state synaptic (but not somatic) levels of CaMKII (Chen et al., 2022), it is difficult to differentiate between the necessity for CaMKII protein to maintain a fully plastic synapse or the requirement for CaMKII local synthesis in the induction of plasticity.

Our results indicate that local CaMKII synthesis is not required for at least one form of structural plasticity-GB formation at the *Drosophila* NMJ. This contrasts sharply with the clear requirement for new CaMKII synthesis in activity-dependent potentiation of spontaneous release rate at this same synapse (Kuklin et al., 2017), suggesting that distinct forms of long-term synaptic plasticity can differ in their dependence on newly synthesized versus preexisting CaMKII. We show that neither pre- nor postsynaptic-specific ablation of the 3′UTR of the endogenous CaMKII locus, nor blockage of the upstream pathways that lead to CaMKII translation, influences activity-induced bouton budding. However, depletion of CaMKII protein on both sides of the synapse leads to deficient outgrowth, pointing to a role of CaMKII protein, but not synthesis, on plasticity. In addition, cell-specific deletion of the coding regions of the *CaMKII* gene demonstrates a requirement for post- but not presynaptic CaMKII.

It is not yet clear how whole-animal CaMKII deficiency prevents activity-dependent GB budding. Although our results show that CaMKII protein is required, it is not clear whether it is due to the enzymatic activity of the kinase or to another, possibly structural function of CaMKII (cf. Tullis et al., 2023; Chen et al., 2024). Another possibility is that the synapses or health of the null animals are simply not normal, either due to a developmental defect or a requirement for CaMKII-dependent synaptic plasticity, e.g., secondary to loss of plasticity of spontaneous release (Kuklin et al., 2017).

Our findings indicate NMJs lacking presynaptic CaMKII protein due to a loss of either the coding region or the 3′UTR have normal GB formation, conflicting with previous results using presynaptically expressed *CaMKII* RNAi, which indicated a dependence on motor neuron CaMKII (Nesler et al., 2016). This difference may derive from the genetic methods used, i.e., targeting the endogenous *CaMKII* locus versus UAS-mediated expression of an RNAi. It is possible that these different manipulations affect protein quantity in synaptic and nonsynaptic compartments differently. *CaMKII* null larvae possess some maternally derived mRNA (Kuklin et al., 2017), which may supply sufficient nonsynaptic protein for GB formation. The discordance with the RNAi results could also reflect differences in the timing of the experimental protocols. We used a protocol from Ataman et al. (2008) which calls for fixation after a 15 min rest period following the final stimulation. In contrast, the presynaptic RNAi study (Nesler et al., 2016) fixed preparations 74 min after the last stimulation. Because GBs are highly dynamic structures, it is quite possible that the two protocols are assaying different biological events. Under this interpretation, presynaptic CaMKII would not be required for the initial formation of protein synthesis-dependent GBs but instead for their subsequent stabilization, such that boutons formed normally but failed to persist until the later fixation time used by Nesler et al. (2016).

An additional interesting result from this study is that NMJs lacking CaMKII in the muscle cell have blunted GB formation. Synaptic growth at the NMJ has been shown to be dependent on retrograde signals, such as members of the bone morphogenetic protein (BMP) pathway, released from the muscle (Yoshihara et al., 2005; Piccioli and Littleton, 2014). While BMP has not been shown to be a direct target of CaMKII, retrograde BMP signaling and postsynaptic CaMKII activity have been linked to homeostatic potentiation at the NMJ (Haghighi et al., 2003). When postsynaptic CaMKII is depleted, BMP or another yet-to-be-identified retrograde pathway may be dysregulated, leading to reduced bouton formation. Another interpretation is that postsynaptic CaMKII may be involved in the remodeling of the muscle cytoskeleton to make way for new presynaptic boutons. Besides GB formation requiring both transsynaptic and intracellular signaling (Yoshihara et al., 2005; Korkut et al., 2013; Piccioli and Littleton, 2014), it is also a mechanical process. Muscle contraction promotes efficient outgrowth (Fernandes et al., 2023), possibly through “pulling” the synaptic membrane during depolarization (Piccioli and Littleton, 2014). Among its many functions, CaMKII can regulate actin and microtubule dynamics (Andersen et al., 2005; Olliff and Sundaramoorthy, 2026), so muscle lacking CaMKII protein may be unable to reorganize the cytoskeleton to accommodate new bouton formation.

In conclusion, our findings demonstrate that activity-dependent GB formation relies on steady-state CaMKII protein expression rather than local translation of this kinase. By manipulating the endogenous *CaMKII* locus, we separated the requirement for CaMKII protein from the requirement for new synthesis by demonstrating that neither pre- nor postsynaptic ablation of the *CaMKII* 3′UTR impairs GB budding. Instead, our data indicate that CaMKII protein itself is required and point to a role for postsynaptic CaMKII, potentially acting through retrograde signaling pathways or regulation of the muscle cytoskeleton. Additional studies will be necessary to define the specific contributions of pre- and postsynaptic CaMKII to activity-dependent bouton sprouting. Moreover, the requirement for new protein synthesis in this form of plasticity appears not to arise from local production of CaMKII, but instead from the production of other as-yet-unidentified factors essential for GB formation. One possible interesting candidate is cortactin, which is present pre- and postsynaptically and has been shown both to increase after patterned stimulation and to be required for GB formation (Alicea et al., 2017).

## Synthesis

Reviewing Editor: Timothy Jarome, Virginia Polytechnic Institute and State University Decisions are customarily a result of the Reviewing Editor and the peer reviewers coming together and discussing their recommendations until a consensus is reached. When revisions are invited, a fact-based synthesis statement explaining their decision and outlining what is needed to prepare a revision will be listed below. The following reviewer(s) agreed to reveal their identity: Mani Ramaswami.

This manuscript represents a well-designed series of experiments that reach an interesting and significant conclusion of CaMKII not being necessary for the activity-dependent formation of ghost boutons During discussion, it was determined that those data presented here make an important contribution to the field. However, several edits were needed before the manuscript is ready for publication

1. It would be useful if past observations on activity-dependent translation of CaMKII at the NMJ be clearly communicated early in the manuscript. It would be useful to state early in the text that prior work has reported spaced stimulation induced CaMKII mRNA translation in the presynapse and if work exists for this in the postsynapse. The current text refers vaguely to "synapse," which is not enough information to frame the questions being asked here.

2. As long-UTRs are often used to both repress basal translation and for subsequent signal-regulated increases in translation (as well as for mRNA localization), it was unclear on how the no-UTR mutant forms of CaMKII affected somatic and synaptic levels of the protein. The current conclusions would be more convincing if the authors directly validated CaMKII depletion or 3′UTR deletion in the relevant tissues for the key DelUTR and DelCoding genotypes in the ghost bouton assay conditions

3. While the paper is clear and deep in its discussion of all prior work on ghost boutons, it would be useful for the reader to have some discussion that considers all ways in which CaMKII and its local translation may contribute to NMJ plasticity. For instance, previous work (Kuklin et al, 2017) showed translation and the CaMKII UTR is needed for potentiation of spontaneous release. This, and a few other prior observations, could be considered in broader and more didactic discussion. The authors should also moderate broad claims about structural plasticity and local protein synthesis, emphasizing instead that their data argue against a requirement for CaMKII local translation in early activity-dependent ghost bouton formation while supporting a role for postsynaptic/muscle CaMKII protein.

4. Minor: Do larvae with muscles or neurons carrying null mutations (or coding sequence deletions) for CaMKII crawl relatively normally? At a superficial level?

## Author Response

This manuscript represents a well-designed series of experiments that reach an interesting and significant conclusion of CaMKII not being necessary for the activity-dependent formation of ghost boutons During discussion, it was determined that those data presented here make an important contribution to the field. However, several edits were needed before the manuscript is ready for publication We thank the reviewers for the fast and positive response to our study. We have made changes in the manuscript that we feel address all the issues raised, and these edits have improved the paper substantially.

1. It would be useful if past observations on activity-dependent translation of CaMKII at the NMJ be clearly communicated early in the manuscript. It would be useful to state early in the text that prior work has reported spaced stimulation induced CaMKII mRNA translation in the presynapse and if work exists for this in the postsynapse. The current text refers vaguely to "synapse," which is not enough information to frame the questions being asked here.

We thank the reviewers for this suggestion. We have expanded the Introduction to outline the roles of CaMKII in NMJ development and plasticity and to discuss its local synthesis at the NMJ to motivate the study more clearly.

2. As long-UTRs are often used to both repress basal translation and for subsequent signal-regulated increases in translation (as well as for mRNA localization), it was unclear on how the no-UTR mutant forms of CaMKII affected somatic and synaptic levels of the protein. The current conclusions would be more convincing if the authors directly validated CaMKII depletion or 3′UTR deletion in the relevant tissues for the key DelUTR and DelCoding genotypes in the ghost bouton assay conditions Previous studies have shown that deletion of the CaMKII 3'UTR reduces CaMKII levels in axons in both adult Kenyon cells (Chen et al., 2022) and motor neurons at the third instar NMJ (Clements et al., 2025). We cite these papers in the revised manuscript. For muscle deletion of the UTR and for deletion of the coding region in muscles and motor neurons, we have added data showing that CaMKII levels are reduced. These have been added as Extended Data files for the appropriate figures.

3. While the paper is clear and deep in its discussion of all prior work on ghost boutons, it would be useful for the reader to have some discussion that considers all ways in which CaMKII and its local translation may contribute to NMJ plasticity. For instance, previous work (Kuklin et al, 2017) showed translation and the CaMKII UTR is needed for potentiation of spontaneous release. This, and a few other prior observations, could be considered in broader and more didactic discussion. The authors should also moderate broad claims about structural plasticity and local protein synthesis, emphasizing instead that their data argue against a requirement for CaMKII local translation in early activity-dependent ghost bouton formation while supporting a role for postsynaptic/muscle CaMKII protein.

We have made a number of changes in response to this comment:

1. We revised the Abstract to restrict our conclusions to the specific process examined here: early activity-dependent ghost bouton formation. We no longer generalize these findings to structural plasticity or local protein synthesis more broadly.

2. As noted in our response to Comment 1, we expanded the Introduction to provide a broader overview of the roles of CaMKII at the larval NMJ, including its presynaptic, postsynaptic, and retrograde functions, and to more clearly summarize prior evidence for activity-dependent local CaMKII synthesis. This provides a broader context for the specific question addressed in our study.

3. We expanded the Discussion to include the findings of the Kuklin study, which demonstrated a requirement for the CaMKII 3′UTR and new CaMKII synthesis in activity-dependent increases in spontaneous release at the same synapse. We now explicitly contrast that form of functional plasticity with the early structural plasticity examined here. This comparison emphasizes that different forms of NMJ plasticity can differ in their requirements for newly synthesized versus pre-existing CaMKII.

4. Minor: Do larvae with muscles or neurons carrying null mutations (or coding sequence deletions) for CaMKII crawl relatively normally? At a superficial level? For the null mutant, the original publication (Kuklin et al., 2017) states that there is 16% lethality during the larval stages and that correlates with our observations that many of the null animals are smaller and less healthy looking. In contrast, while we have not looked at this quantitatively, we not did see any obvious differences in viability, size or activity when cell-specific alterations were made in the gene.
