# Supplementary material for: Local Synthesis of CaMKII Does Not Contribute to Activity-Dependent Generation of Ghost Boutons
Source: eNeuro. 2026 Sep 11;13(9):ENEURO.0196-26.2026. doi: 10.1523/ENEURO.0196-26.2026 (PMC13583335; doi:10.1523/ENEURO.0196-26.2026)
Supplement: Supplementary file 1 [file eneuro-13-ENEURO.0196-26.2026-s004.docx]

| Figure | **Measurement** | **Genotype** | **Statistical test** | **Post hoc test** | **Comparison** | **Summary** | **p value** | **Sample sizes** |
| --- | --- | --- | --- | --- | --- | --- | --- | --- |
| 2A | # Ghost boutons/NMJ | Canton S | 2 Way ANOVA | Šídák's multiple comparisons | Axon uncut:Norm vs. Axon uncut:K+ | **** | <0.0001 | 8, 8 |
|  |  |  |  |  | Axon cut:Norm vs. Axon cut:K+ | * | 0.0369 | 8, 8 |
|  |  |  |  |  | Axon uncut:Norm vs. Axon cut:Norm | ns | 0.9254 | 8, 8 |
|  |  |  |  |  | Axon uncut:K+ vs. Axon cut:K+ | ns | 0.3386 | 8, 8 |
| 3A | # Ghost boutons/NMJ | Frt-Stop-Frt-Halo-CaMKII/EGFP (+/-) or Frt-Stop-Frt-Halo-CaMKII/Frt-Stop-Frt-Halo-CaMKII (-/-) | 2 Way ANOVA | Šídák's multiple comparisons | +/-:Norm vs. +/-:K+ | **** | <0.0001 | 14, 14 |
|  |  |  |  |  | +/-:Norm vs. +/-:K+ | ns | 0.9825 | 14, 16 |
|  |  |  |  |  | -/-:Norm vs. -/-:K+ | ns | 0.9927 | 16, 13 |
|  |  |  |  |  | +/-:K+ vs. -/-:K+ | **** | <0.0001 | 14,13 |
| 3B | CaMKII intensity | Frt-Stop-Frt-Halo-CaMKII/EGFP (+/-) or Frt-Stop-Frt-Halo-CaMKII/Frt-Stop-Frt-Halo-CaMKII (-/-) | 2 Way ANOVA | Šídák's multiple comparisons | +/-:Presynaptic vs. +/-:Postsynaptic | **** | <0.0001 | 16,16 |
|  |  |  |  |  | +/-:Presynaptic vs. -/-:Presynaptic | **** | <0.0001 | 16,16 |
|  |  |  |  |  | -/-:Presynaptic vs. -/-:Postsynaptic | ns | 0.1902 | 16,16 |
|  |  |  |  |  | +/-:Postsynaptic vs. -/-:Postsynaptic | **** | <0.0001 | 16,16 |
| 4A | # Ghost boutons/NMJ | Flp Ctrl, C164 Ctrl, Pre DelUTR | 2 Way ANOVA | Šídák's multiple comparisons | Flp ctrl:Norm vs. Flp ctrl:K+ | *** | 0.0002 | 16,16 |
|  |  |  |  |  | C164 ctrl:Norm vs. C164 ctrl:K+ | ** | 0.0057 | 16,16 |
|  |  |  |  |  | DelUTR (pre):Norm vs. DelUTR (pre):K+ | **** | <0.0001 | 16,15 |
|  |  |  |  |  | Flp ctrl:K+ vs. DelUTR (pre):K+ | ns | 0.9996 | 16,15 |
|  |  |  |  |  | C164 ctrl:K+ vs. DelUTR (pre):K+ | ns | 0.4941 | 16,15 |
| 4B | # Ghost boutons/NMJ | Flp Ctrl, MHC Ctrl, Post DelUTR | 2 Way ANOVA | Šídák's multiple comparisons | Flp ctrl:Norm vs. Flp ctrl:K+ | ** | 0.0014 | 13, 15 |
|  |  |  |  |  | MHC ctrl:Norm vs. MHC ctrl:K+ | **** | <0.0001 | 14, 13 |
|  |  |  |  |  | Post DelUTR:Norm vs. Post DelUTR:K+ | *** | 0.0003 | 15, 16 |
|  |  |  |  |  | Flp ctrl:K+ vs. Post DelUTR:K+ | ns | 0.7548 | 15, 16 |
|  |  |  |  |  | MHC ctrl:K+ vs. Post DelUTR:K+ | ns | 0.9989 | 13, 16 |
| 5A | # Ghost boutons/NMJ | Canton S | Mann Whitney (two tailed) |  | Norm vs K+ | **** | <0.0001 | 14,15 |
| 5B | # Ghost boutons/NMJ | T287T, T287A | 2 Way ANOVA | Šídák's multiple comparisons | T287T:Norm vs. T287T:K+ | *** | 0.0002 | 16,15 |
|  |  |  |  |  | T287T:Norm vs. T287A:Norm | ns | 0.9983 | 16,14 |
|  |  |  |  |  | T287T:K+ vs. T287A:K+ | **** | <0.0001 | 15,15 |
|  |  |  |  |  | T287A:Norm vs. T287A:K+ | **** | <0.0001 | 14,15 |
| 5C | # Ghost boutons/NMJ | T287T, T287A,T306/7 | 2 Way ANOVA | Šídák's multiple comparisons | T287T:Norm vs. T287T:K+ | **** | <0.0001 | 16, 15 |
|  |  |  |  |  | T287A,T306/7AA:Norm vs. T287A,T306/7AA:K+ | **** | <0.0001 | 14, 15 |
|  |  |  |  |  | T287T:K+ vs. T287A,T306/7AA:K+ | ns | 0.9059 | 15, 15 |
|  |  |  |  |  | T287T:Norm vs. T287A,T306/7AA:Norm | ns | 0.9779 | 16,14 |
| 6A | # Ghost boutons/NMJ | Flp Ctrl, C164 Ctrl, Pre DelCoding | 2 Way ANOVA | Šídák's multiple comparisons | Flp ctrl:Norm vs. Flp ctrl:K+ | ns | 0.0649 | 15, 13 |
|  |  |  |  |  | C164 ctrl:Norm vs. C164 ctrl:K+ | *** | 0.0003 | 15, 13 |
|  |  |  |  |  | DelCoding (Pre):Norm vs. DelCoding (Pre):K+ | **** | <0.0001 | 16, 16 |
|  |  |  |  |  | Flp ctrl:K+ vs. DelCoding (Pre):K+ | ns | 0.2758 | 13, 16 |
|  |  |  |  |  | C164 ctrl:K+ vs. DelCoding (Pre):K+ | ns | 0.9946 | 13, 16 |
| 6B | # Ghost boutons/NMJ | Flp Ctrl, MHC Ctrl, Post DelCoding | 2 Way ANOVA | Šídák's multiple comparisons | Flp ctrl:Norm vs. Flp ctrl:K+ | **** | <0.0001 | 19, 16 |
|  |  |  |  |  | MHC ctrl:Norm vs. MHC ctrl:K+ | **** | <0.0001 | 16, 16 |
|  |  |  |  |  | Post DelCoding:Norm vs. Post DelCoding:K+ | ns | 0.3539 | 12, 20 |
|  |  |  |  |  | Flp ctrl:K+ vs. Post DelCoding:K+ | ** | 0.0023 | 16, 20 |
|  |  |  |  |  | MHC ctrl:K+ vs. Post DelCoding:K+ | * | 0.0148 | 16, 20 |
| 4.1B | Postsynaptic CaMKII intensity | Flp Ctrl, MHC Ctrl, Post DelUTR | 2 Way ANOVA | Šídák's multiple comparisons | Flp ctrl Norm vs. K+ | ** | 0.0011 | 12, 10 |
|  |  |  |  |  | MHC Ctrl Norm vs. K+ | ** | 0.0026 | 12, 11 |
|  |  |  |  |  | Post DelUTR Norm vs. K+ | ns | 0.0828 | 11, 12 |
|  |  |  |  |  | Norm |  |  |  |
|  |  |  |  |  | Flp ctrl vs. MHC ctrl | ns | 0.8958 | 12, 12 |
|  |  |  |  |  | Flp ctrl vs. DelUTR (post) | ns | 0.9204 | 12, 11 |
|  |  |  |  |  | MHC ctrl vs. DelUTR (post) | ns | 0.5587 | 12, 11 |
|  |  |  |  |  | K+ |  |  |  |
|  |  |  |  |  | Flp ctrl vs. MHC ctrl | ns | 0.9948 | 10, 11 |
|  |  |  |  |  | Flp ctrl vs. DelUTR (post) | ns | 0.0779 | 10, 12 |
|  |  |  |  |  | MHC ctrl vs. DelUTR (post) | * | 0.0383 | 11, 12 |
| 6.1B | Presynaptic CaMKII intensity | Flp Ctrl, C164 Ctrl, Pre DelCoding | 2 Way ANOVA | Šídák's multiple comparisons | Flp ctrl Norm vs. K+ | *** | 0.0004 | 16, 12 |
|  |  |  |  |  | C164 Ctrl Norm vs. K+ | ** | 0.0046 | 15, 11 |
|  |  |  |  |  | Pre DelCoding Norm vs. K+ | ns | 0.8473 | 16, 16 |
|  |  |  |  |  | Norm |  |  |  |
|  |  |  |  |  | Flp ctrl vs. C164 ctrl | ns | 0.4764 | 16, 15 |
|  |  |  |  |  | Flp ctrl vs. DelCoding (Pre) | **** | <0.0001 | 16, 16 |
|  |  |  |  |  | C164 ctrl vs. DelCoding (Pre) | **** | <0.0001 | 15, 16 |
|  |  |  |  |  | K+ |  |  |  |
|  |  |  |  |  | Flp ctrl vs. C164 ctrl | ns | 0.9468 | 12, 11 |
|  |  |  |  |  | Flp ctrl vs. DelCoding (Pre) | **** | <0.0001 | 12, 16 |
|  |  |  |  |  | C164 ctrl vs. DelCoding (Pre) | **** | <0.0001 | 11, 16 |
| 6.1D | Postsynaptic CaMKII intensity | Flp Ctrl, MHC Ctrl, Post DelCoding | 2 Way ANOVA | Šídák's multiple comparisons | Flp ctrl Norm vs. K+ | **** | <0.0001 | 15, 15 |
|  |  |  |  |  | MHC ctrl Norm vs. K+ | **** | <0.0001 | 11, 10 |
|  |  |  |  |  | Post DelCoding Norm vs. K+ | ns | 0.1547 | 12, 14 |
|  |  |  |  |  | Norm |  |  |  |
|  |  |  |  |  | Flp vs. MHC | ns | 0.8782 | 15, 11 |
|  |  |  |  |  | Flp vs. Post DelCoding | **** | <0.0001 | 15, 12 |
|  |  |  |  |  | MHC vs. Post DelCoding | *** | 0.0003 | 11, 12 |
|  |  |  |  |  | K+ |  |  |  |
|  |  |  |  |  | Flp vs. MHC | ns | 0.2408 | 15, 10 |
|  |  |  |  |  | Flp vs. Post DelCoding | **** | <0.0001 | 15, 14 |
|  |  |  |  |  | MHC vs. Post DelCoding | **** | <0.0001 | 10, 14 |

**Figure 1-1. Statistical analyses and genotypes.**
